# A novel anti-human CD25 mAb with preferential reactivity to activated T regulatory cells depletes them from the tumor microenvironment

**DOI:** 10.18632/oncotarget.28752

**Published:** 2025-07-09

**Authors:** Maja Buszko, Madalyn Jones, Sruthi Chempati, Lyra Morina, Kirstin Ward, Habtom Habte, Michael Dziegielewski, Ethan M. Shevach

**Affiliations:** ^1^Laboratory of Immune System Biology, National Institute of Allergy and Infectious Diseases, Bethesda, MD 20892, USA; ^2^Boehringer Ingelheim, Ridgefield, CT 06877, USA

**Keywords:** T_reg_, CD25, TME, mAb, GVHD

## Abstract

T_reg_ play a deleterious role in the tumor microenvironment by suppressing anti-tumor effector T cells. Deletion of T_reg_ can result in an enhanced anti-tumor response. It has been difficult to identify a cell surface antigen that is uniquely expressed on T_reg_ which can be targeted by a deleting mAb. We immunized mice with human T_reg_ cells which had been activated and expanded *in vitro*. One hybridoma (2B010) which recognized CD25 was identified. 2B010 demonstrated selective reactivity to T_reg_ cells that had been expanded in culture for 5 days, but displayed similar reactivity to a conventional anti-CD25 mAb on freshly expanded T_reg_. 2B010 did not block the binding of IL-2 in the STAT5 phosphorylation assay and had no effect on the proliferation of T_conv_ or on T_reg_ suppressor function. It selectively reacted with T_reg_ activated *in vivo* during xeno-GVHD and produced a selective deletion of T_reg_ from mice undergoing xeno-GVHD. Administration of 2B010 to tumor bearing humanized mice resulted in a profound depletion of T_reg_ from the TME and activation of CD8^+^ T cells. No effect on tumor growth was observed. 2B010 represents a candidate for treatment of patients with cancer either alone or together with check point inhibitors.

## INTRODUCTION

While T regulatory cells (T_reg_) play a beneficial role in maintaining immune homeostasis and preventing the induction of autoimmune disease, T_reg_ play a deleterious role in the tumor microenvironment (TME) by inhibiting the function of activated CD4^+^ and CD8^+^ T conventional (T_conv_) cells [1–4]. Infiltration of T_reg_ into the TME has been shown to correlate with severity of the tumor stage and poor prognosis [5]. One of the major problems in the evaluation of the contribution of T_reg_ to the inhibition of T_conv_ cell function in the TME is the lack of a monoclonal antibody (mAb) to a cell surface antigen capable of specifically recognizing and deleting resting/activated T_reg_ cells or reversing their suppressive function. Several members of the tumor necrosis receptor family superfamily [6] (TNFRSF) including TNFR2 [7], GITR [8], OX-40 [9], and 4-1BB [10, 11], chemokine receptors (CCR4 [12], CCR8 [13]) and co-inhibitory receptors (CTLA-4 [14–17], PD-1 [18, 19], LAG-3 [20], and Tim-3 [21]) appear to have enhanced expression on intra-tumoral T_reg._ While mAbs to some of these targets (CTLA-4, PD-1, CCR4 and CCR8) have anti-tumor effects by acting on Treg, the other targets are frequently expressed on activated T_conv_ populations and have not yet proven useful for the augmentation of tumor immunity. The expression of CD25, the IL-2R α-chain, was originally used to define T_reg_ cells and expression of CD25 correlates well with the expression of Foxp3 [22]. Even though CD25 is expressed by any activated T cell [23], the levels of expression of CD25 on T_reg_ in the TME appear to be much higher than the levels of CD25 on T_conv_ in the TME. Recently, an anti-CD25 mAb which does not block the binding of IL-2 to its receptor complex and is optimized for antibody dependent cellular cytotoxicity (ADCC)- and antibody dependent cellular phagocytosis (ADCP)- has shown promise in pre-clinical studies [24] and has recently entered phase I clinical trials [25].

In this study, we have taken an unbiased approach to the search for a unique T_reg_ cell surface molecule that could be potentially used to delete cells from the TME or to reverse the suppressive function of T_reg_. We used several different protocols to generate mAbs in mice that had been immunized with polyclonal expanded sorted human T_reg_ cells using both standard and rapid immunization protocols as well as tolerization of mice to human cell surface antigens that were not expressed on T_reg_. Our major screening technique was to identify mAbs that would bind to activated T_reg_ but would not bind to activated CD4^+^ T cells that had been expanded *in vitro* for a comparable time using similar stimulation conditions. Surprisingly, we identified several mAbs that recognized CD25 and allowed for the best discrimination between expanded Treg and expanded CD4^+^Foxp3^−^ Teff cells. One of these mAbs (2B010) was studied in detail and shown to preferentially delete activated T_reg_ cells from mice with xenogeneic graft versus host disease (xeno-GVHD) [26], while preserving activated T_conv_ cell numbers and function. In addition, this mAb was capable of markedly deleting T_reg_ from the TME of humanized mice that had been injected orthotopically with a human breast cancer cell line. Taken together, these pre-clinical studies suggest that mAb 2B010 has the potential to move on to clinical trials in humans.

## RESULTS

### Characterization of mAb 2B010

**Figure 1:**
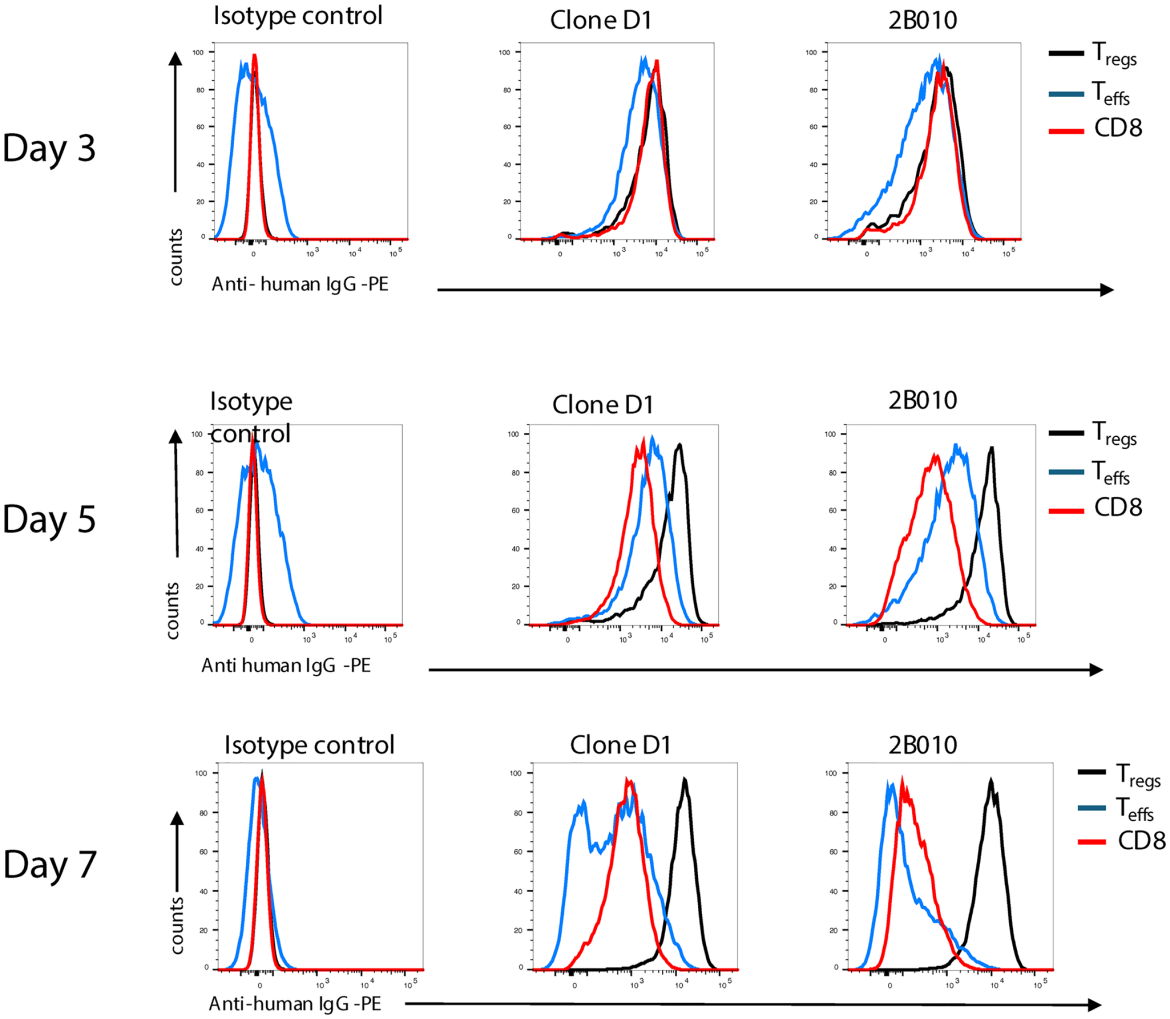
2B010 preferentially reacts with T_reg_ expanded *in vitro*. CD4^+^CD25^+^, CD4^+^CD25^−^, and CD8^+^ T cells were isolated from human PBMCS by cell sorting and stimulated with anti-CD3/CD28 beads and IL-2. Cells were stained with Clone D1 or 2B010 on days 3, 5 and 7 after stimulation. One representative example of three different donors is shown.

The initial goal of these studies was to develop mAbs that specifically recognized human CD4^+^Foxp3^+^ T_reg_ cells but failed to react with naïve or activated CD4^+^Foxp3^−^ T_conv_. We used a rapid immunization strategy in which mice were injected 6 times over a 4-week period with expanded FACS-sorted human T_reg_ (Supplementary Figure 1A). Following fusion, supernatants were screened on expanded T_reg_ and expanded T_conv_. Several of the mouse mAbs generated reacted preferentially with expanded T_reg_ compared to expanded T_conv_. We generated a chimeric version of one clone (2B010) containing the wild type human IgG_1_ Fc region. 2B010 failed to block the binding of several other mAbs to CD25 when assayed on freshly isolated CD4^+^Foxp3^+^ T cells (Supplementary Figure 2), but blocked its own binding. mAbs BC96 or 2A3 were used in subsequent experiments to assay CD25 expression. 2B010 exhibited the greatest reactivity with T_reg_ but low reactivity with expanded T_conv_ or with expanded CD8^+^ T cells and was selected for further study. In contrast, the daclizumab-like mAb Clone D1, used for the prevention of allograft rejection [27], showed greater reactivity with T_reg_, but still exhibited significant staining of T_conv_ and CD8^+^ T cells (Figure 1, and Supplementary Figure 3A). Similar results were observed with expanded cells from 3 different donors. Although the staining profiles of 2B010 and daclizumab differed significantly, 2B010 stained CHO cells that had been transfected with human CD25, but not un-transfected CHO cells, indicating that 2B010 also recognized CD25 (Supplementary Figure 1B). Curiously, the preferential binding of 2B010 to expanded T_reg_ was only observed following *in vitro* expansion for 7 days or longer and was not seen at shorter time periods after activation when its staining pattern was identical to Clone D1 (Figure 1).

**Figure 2:**
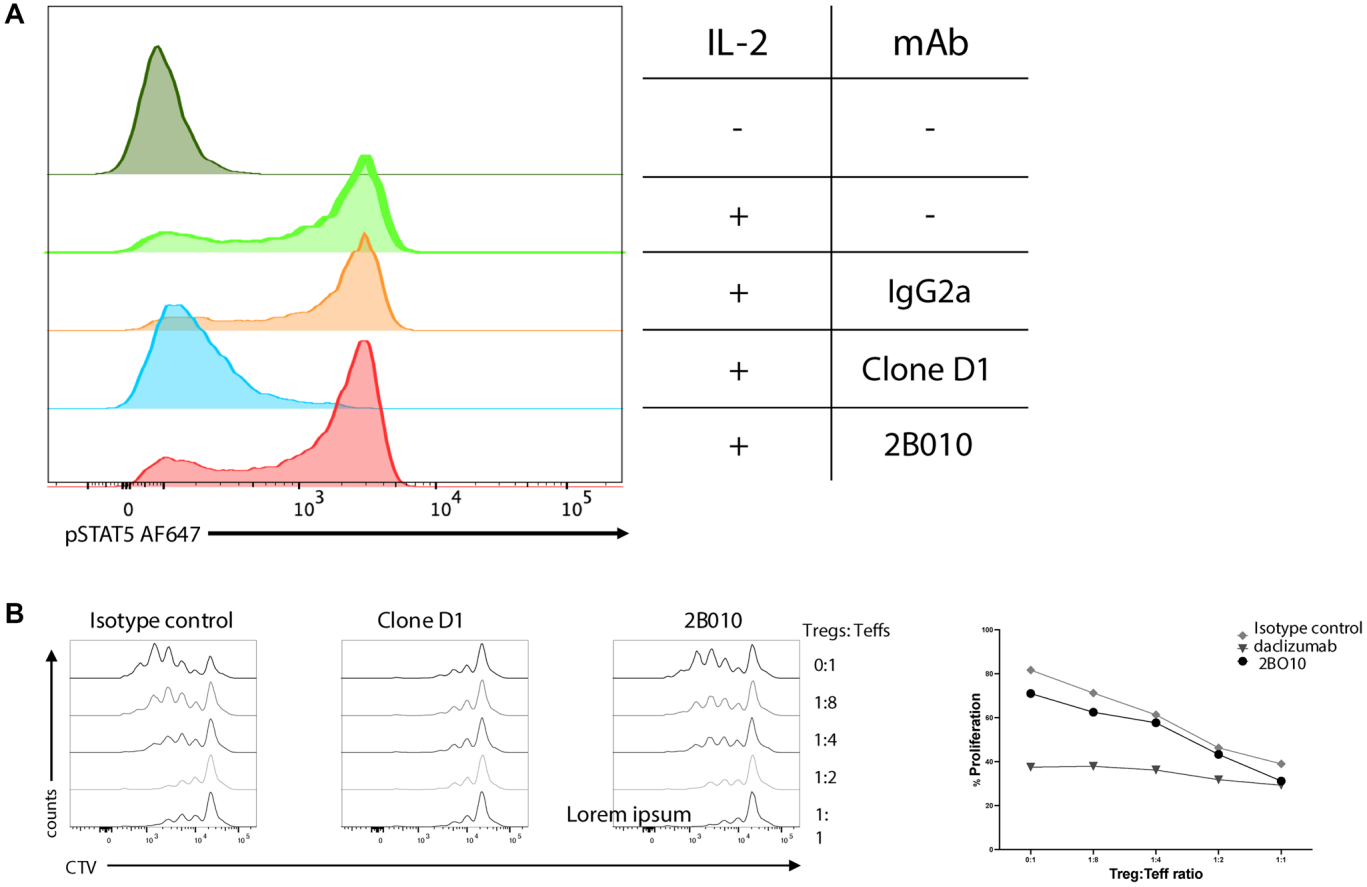
mAb 2B010 does not react with the IL-2 binding site. (**A**) CD4^+^ T cells were purified from PBMC by cell sorting, stimulated with IL-2 for 20 minutes in the presence or absence of isotype control mAb, clone D1, or 2B010. Cells were then washed and stained for pSTAT5. (**B**) CD4^+^CD25^−^ T cells were sorted from PBMCs, labeled with CTV, and then cultured alone or in the presence of cell sorter purified T_reg_ (CD4^+^CD25^+^) at the ratios indicated. All cultures were stimulated with soluble OKT3 (1 μg/ml) and cell sorter purified CD3^−^DR^+^ cells. One representative example of three different donors is shown.

The binding affinity for CD25 was significantly higher for Clone D1 compared to 2B010 (*K*_D_ 9.3X^−12^ M versus *K*_D_ 5.03X^−9^ M, Supplementary Figure 1C) as measured by surface plasmon resonance using the human CD25 extracellular domain. Our further attempts to determine the epitope recognized by mAb 2B010 using SPR were not successful. We then performed epitope mapping by comparing the binding of 2B010 with another previously characterized anti-human CD25 mAb, basiliximab, which recognizes the IL-2 binding site on CD25 [28]. Basiliximab failed to inhibit the binding of 2B010. Similar results were seen with clone MA251 which has previously been shown to bind to a site unrelated to the IL-2 binding site on CD25. In contrast, the binding of clone BC96 which has been shown to block IL-2 binding [29] was readily blocked by basiliximab (Supplementary Figure 1D). One of the earliest events following the binding of IL-2 to its receptor complex is phosphorylation of STAT5. While Clone D1 blocked STAT5 phosphorylation, phosphorylation was not impacted in the presence of 2B010 confirming that mAb 2B010 recognized a site on CD25 distinct from the IL-2 binding site (Figure 2A and online Supplementary Figure 3B). 2B010 had no effect on the proliferation of CD4^+^ T cells by anti-human CD3ε mAb (clone OKT3), while significant inhibition of T cell activation was produced by clone D1 (Figure 2B). 2B010 also had no effects on T_reg_ mediated suppression of T cell proliferation (Figure 2B).

### 2B010 selectively reacts with activated T_reg_ *in vivo*

**Figure 3:**
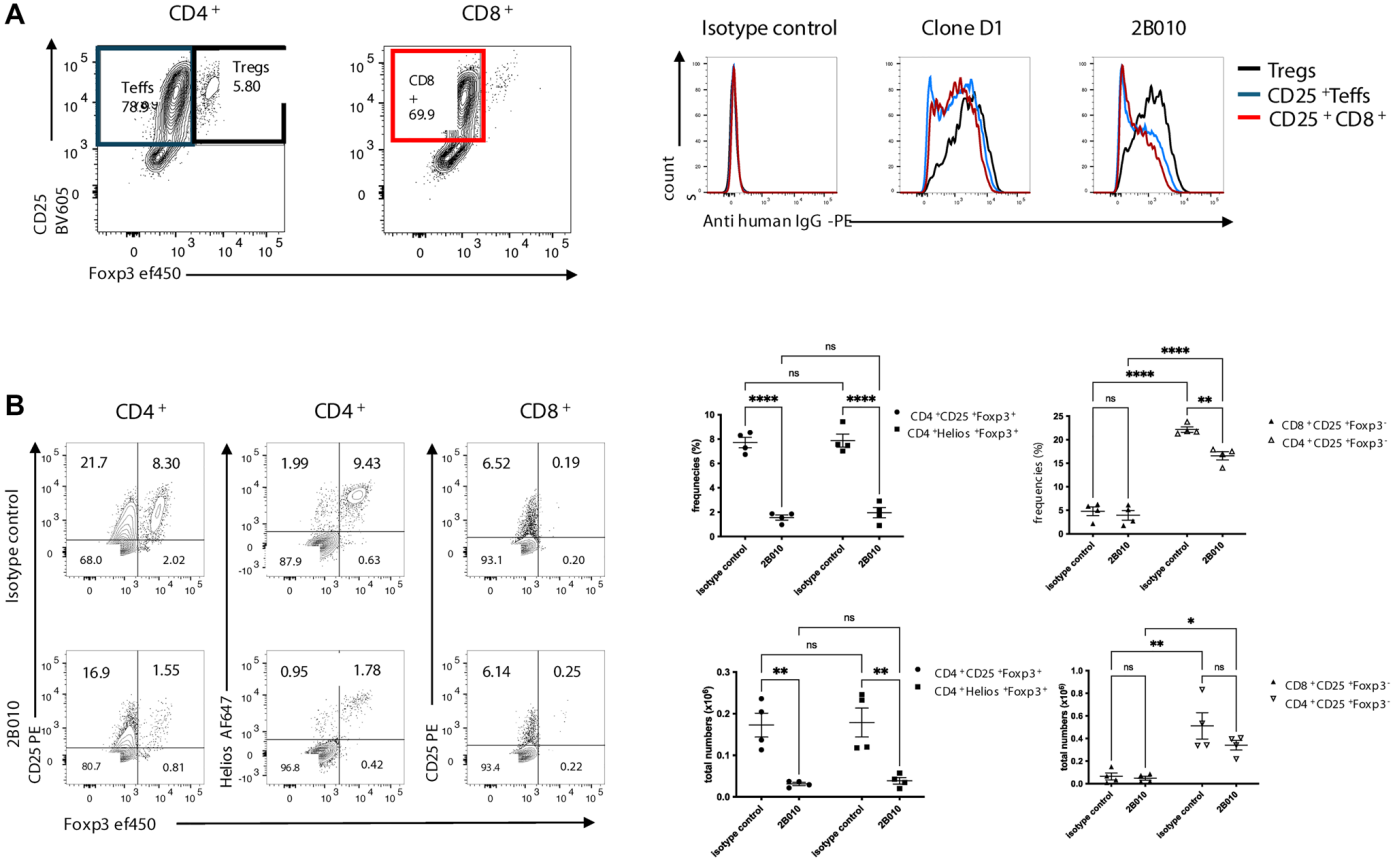
2B010 preferentially reacts with and depletes activated T_reg_ *in vivo* during xeno-GvHD. (**A**) NSG mice were injected with hPBMC (3 × 10^6^), spleens were harvested 7 days later and reactivity with Clone D1 and 2B010 was analyzed on T_reg_, T_conv_ and CD25^+^CD8^+^ T cells. (**B**) NSG mice were injected with hPBMC (3 × 10^6^) and 14 days later 400 μg of 2B010 or isotype control were injected intravenously. Mice were euthanized 5 days post treatment and frequencies and total numbers of CD4^+^CD25^+^Foxp3^+^, CD4^+^CD25^+^Helios^+^, CD4^+^CD25^+^Foxp3^−^, and CD8^+^CD25^+^Foxp3^−^ T cells in the spleen were measured. *n* = 4 mice per group, two-way ANOVA with Tukey test was used for the analysis. Data are shown as mean ± SEM. One representative sample of three separate experiments is shown.

One potential problem with the *in vitro* studies demonstrating enhanced staining of expanded T_reg_ by 2B010 is that the expansion of T_reg_ *in vitro* is dependent on the addition of exogenous IL-2 which in turn may regulate expression of CD25 [30]. We therefore used a model in which human peripheral blood mononuclear cells (PBMCs) are adoptively transferred to immunodeficient NSG mice, resulting in gradual activation of the human T cells which recognize mouse antigens leading to the development of lethal xeno-GVHD after 2–3 months [31]. We harvested spleens from mice that received human PBMC 14 days earlier and compared the reactivity of CD4^+^Foxp3^−^, CD4^+^Foxp3^+^, and CD8^+^ T cells with Clone D1 and 2B010. All three cell populations expressed high levels of CD25 when reacted with Clone D1, but when reacted with 2B010 only T_reg_ expressed high levels of CD25 while much lower levels of CD25 could be detected on CD4^+^Foxp3^−^ and CD8^+^ T cells (Figure 3A). Thus, the selective reactivity of 2B010 with expanded T_reg_ observed *in vitro* could be reproduced in a more physiological environment of T cell activation *in vivo*.

### 2B010 preferentially depletes activated T_reg_ *in vivo* during xeno-GVHD

To assess the potential of 2B010 mediated Treg depletion *in vivo* we transferred human PBMCs to NSG mice and on day 14 after transfer, injected 2B010 or an isotype control mAb. On day 5 after treatment, mice were euthanized by CO2 narcosis and the absolute number and frequencies of CD4^+^CD25^+^Foxp3^−^, CD4^+^CD25^+^Foxp3^+^, and CD8^+^CD25^+^ T cells were determined. Treatment with 2B010 resulted in a marked depletion of CD4^+^CD25^+^Foxp3^+^, but not CD4^+^CD25^+^Foxp3^−^ or CD8^+^CD25^+^ T cells (Figure 3B). To rule out the possibility that the apparent loss of CD4^+^CD25^+^Foxp3^+^ cells was secondary to modulation of CD25 from the cell surface, we also assayed expression of the transcription factor Helios in the CD4^+^CD25^+^Foxp3^+^ after treatment. Helios is expressed by ~80–90% of human Foxp3^+^ T_reg_ and not expressed by either resting or activated T_conv_. The decrease in CD4^+^CD25^+^Foxp3^+^ cells was identical to the decrease in CD4^+^CD25^+^Helios^+^ cells (Figure 3B) confirming the specific depletion of Treg cells. We did observe a small percentage of CD4^+^Foxp3^−^Helios^+^ T cells (Figure 3B). In general, these cells expressed much lower levels of Helios than CD4^+^Foxp3^+^CD25^+^ T cells. These cells may represent Treg that have downregulated Foxp3 expression during the course of the study, but this conclusion is speculative. Antibody-mediated depletion is usually the result of Fc/FcR interactions leading to ADCC. To further confirm that the major *in vivo* function of 2B010 was the depletion of Foxp3^+^CD25^+^ T_reg_, we treated mice with unmodified chimeric 2B010 or 2B010 with a silent Fc region. Marked depletion of CD4^+^CD25^+^Foxp3^+^ T cells was seen in mice treated with wild type 2B010, but no depletion was seen in mice treated with the 2B010 expressing a silent Fc region (Supplementary Figure 4) indicating that depletion is the result of ADCC.

### 2B010 depletes T_reg_ expressing high levels of CD25, but transiently modulates CD25 expression on T_reg_ expressing lower levels of CD25

**Figure 4:**
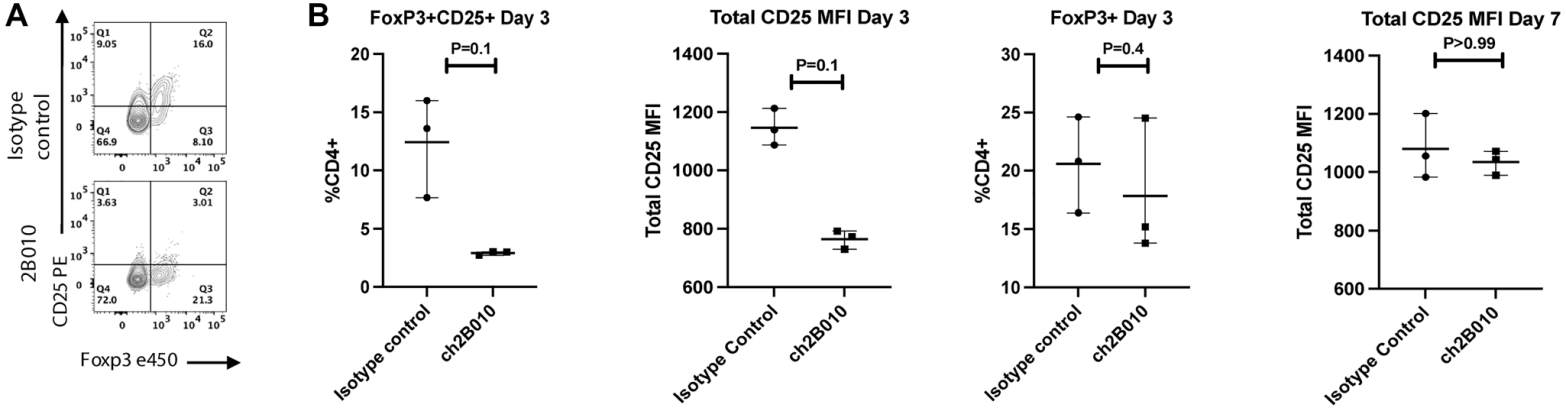
2B010 tends to modulate low levels of CD25 expression. huCD34^+^ NOG EXL mice were treated with 400 μg 2B010 or isotype control and frequencies of T_reg_ were analyzed in the blood over the course of 1 week. *n* = 3 mice per group. (**A**) The results of one representative animal on day 3 after treatment are shown. We gated on total CD4^+^ T cells. (**B**) Summary data of percentage CD4^+^CD25^+^ T cells, CD25 expression as MFI on both CD4^+^Foxp3^−^ and CD4^+^Foxp3^−^ T cells, percentage of CD4^+^Foxp3^+^ T cells on day 3, and CD25 expression as MFI on both CD4^+^Foxp3^−^ and CD4^+^Foxp3^+^ T cells on day 7 after treatment. Mann-Whitney statistical test was used. Data are shown as means ± SEM.

One goal of the therapeutic use of anti-CD25 in the TME would be to deplete highly suppressive T_reg_ that express high levels of CD25 but to spare peripheral T_reg_ that maintain tolerance and immune homeostasis. To determine the effects of 2B010 on T cells that express lower levels of CD25, we analyzed CD25 expression on CD4^+^ T cells in a different humanized mouse model in which immunodeficient NOG mice are reconstituted with CD34^+^ stem cells shortly after birth. Human T and B lymphocytes develop in these mice, but the mice do not succumb to xeno-GVHD as the differentiated human lymphocytes recognize mouse antigens as “self.” The treatment of these mice with 2B010 resulted in a transient loss of CD25 expression on day 3 on both CD4^+^CD25^+^Foxp3^−^ and CD4^+^CD25^+^Foxp3^+^ T cells which did not reach statistical significance. However, the modest loss of CD25 expression on T_reg_ was not secondary to depletion of T_reg_ as the percentage of Foxp3^+^ T cells was unchanged after treatment (Figure 4). The loss of CD25 expression was transient as the levels of CD25 expression were normal on day 7 after treatment. The percentages of CD4^+^CD25^+^Foxp3^+^ as well as CD4^+^Foxp3^+^Helios^+^ T cells on day 7 were also not different from control treated mice (data not shown). Thus, the capacity of 2B010 to deplete T_reg_ appears to be directly related to the level of CD25 expression on T_reg_ *in vivo*.

### 2B010 minimally depletes activated CD4^+^CD25^+^Foxp3^−^ T cells

**Figure 5:**
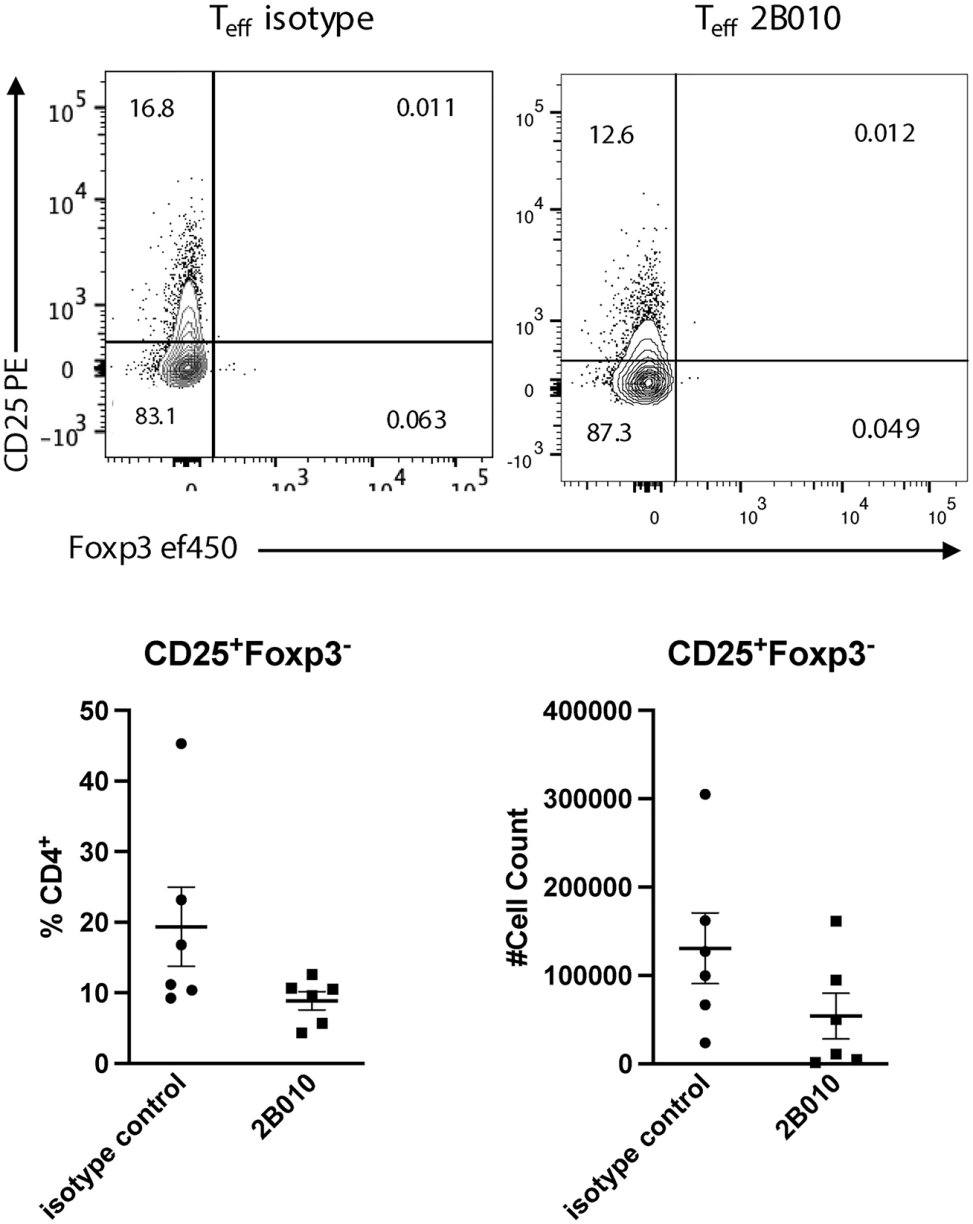
2B010 does not deplete activated CD4^+^CD25^+^Foxp3^−^ T cells *in vivo*. CD4^+^CD25^−^ were sorted from PBMCs, expanded *in vitro* for 4 days in the presence of anti-CD3/CD28 coated beads and IL-2 (100 U/ml) as is Figure 1 and then injected (3 × 10^6^) into NSG mice. One week later, mice were treated with 400 μg isotype control or 2B010 and euthanized 12 days later. Frequencies and absolute numbers of CD4^+^CD25^+^ T cells in the spleens were determined by flow cytometry. Graphs represent summary data of CD4^+^CD25^+^ frequency and cell number. *n* = 6 mice per group. Mann-Whitney statistical test was used, all comparisons are ns. Data are shown as means ± SEM. One representative study of three is shown.

One possibility for the selective depletion of T_reg_ from NSG mice reconstituted with PBMC is that the CD4^+^Foxp3^−^ T cells never achieve a level of CD25 expression that would allow depletion secondary to inhibition of their activation by T_reg_ in the transferred PBMCs. To address this possibility, we transferred *in vitro* expanded CD4^+^Foxp3^−^ T cells (100% CD25+, Figure 1) alone into NSG mice and treated the recipients with 2B010 one week later. When the recipients were analyzed five days later, about 20% of the transferred cells continued to express CD25 in the control treated mice and treatment with 2B010 resulted in a slightly lower but statistically non-significant level of CD25 expression and a similar non-significant decrease in the absolute numbers of CD4^+^Foxp3^−^ T cells recovered (Figure 5). Thus, highly activated CD4^+^Foxp3^−^ T cells appear to be more resistant to depletion by 2B010.

### 2B010 depletes T_reg_ from the TME and spleen in CD34^+^ engrafted NSG mice

**Figure 6:**
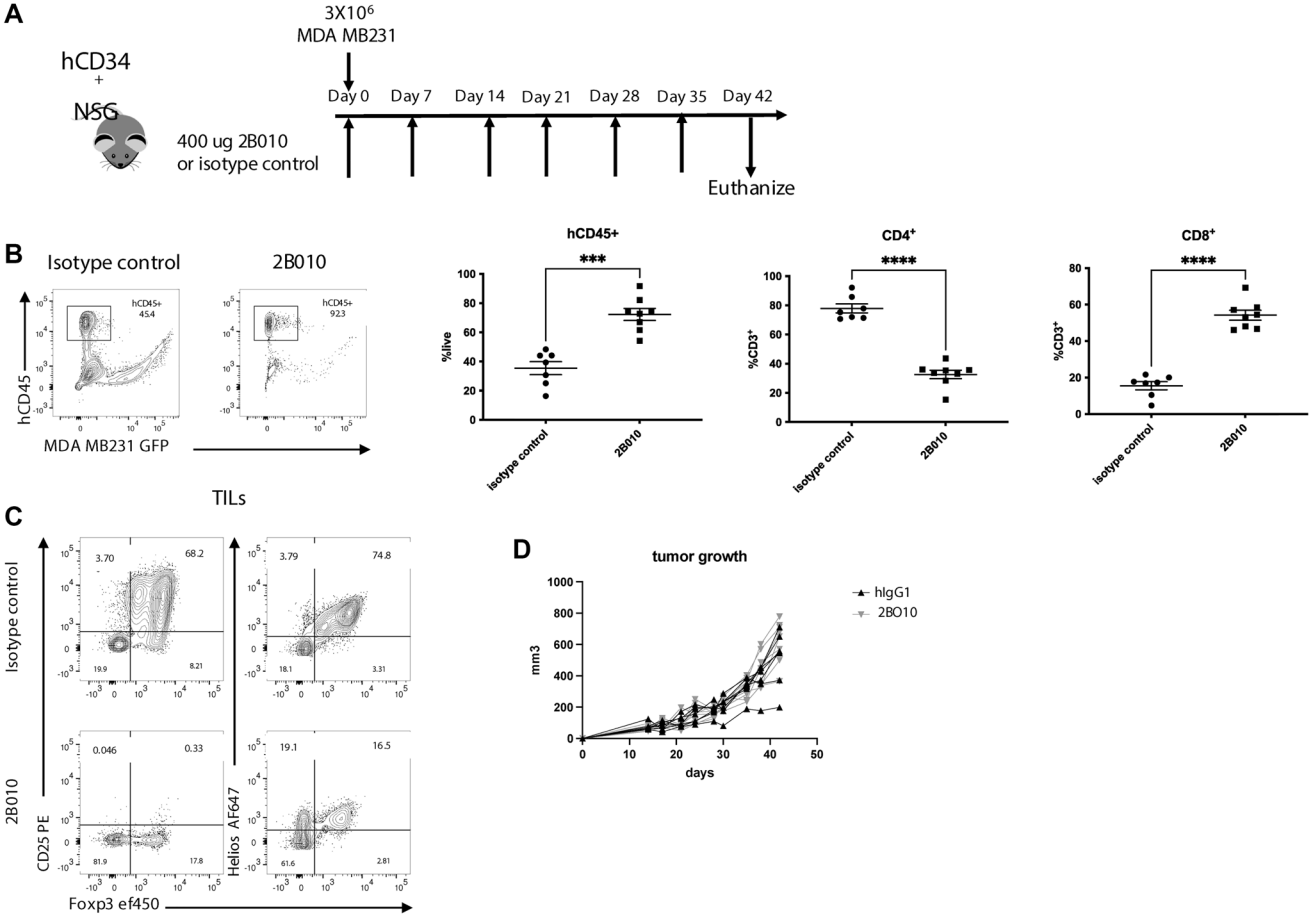
2B010 depletes T_reg_ from the TME in humanized mice. (**A**) huCD34^+^ NSG mice were implanted orthotopically with MDA MB231 – luc/GFP (3 × 10^6^) cells and received 400 μg of 2B010 or isotype control weekly for a total of 6 doses. At the end of the study (day 42) TILs and spleens were harvested and analyzed by flow cytometry. (**B**) Frequencies of human CD45^+^, CD4^+^ and CD8^+^ T cells in the TIL. (**C**) Flow cytometry plots showing CD25 and Helios expression on CD4^+^ T cells isolated from TILs. (**D**) Graph showing tumor growth in the 2B010 and isotype control groups. *n* = 7–8 mice per group. Mann-Whitney statistical test was used. Data are shown as means ± SEM. This experiment was done once.

Since Treg play an important role in immunosuppression in the TME, we next evaluated the capacity of 2B010 treatment to deplete Treg both in the TME and peripherally in the spleen. CD34^+^ stem cell reconstituted NSG mice were implanted orthotopically with 3 × 10^6^ MDA MB231– luc/GFP breast cancer cells and immediately treated with 2B010 or isotype control weekly for total of 6 doses. On day 42 after tumor implantation, tumor infiltrating lymphocytes (TILs) and spleens were harvested and analyzed by flow cytometry (Figure 6A). A significant number of human CD45^+^ cells were detected in the TIL and CD4^+^ T cells were dominant in the isotype-treated control animals, while CD8^+^ T cells predominated in the 2B010 treated mice (Figure 6B). Surprisingly, the majority of the CD4^+^ T cells in the TIL of control mice were Foxp3^+^Helios^+^ T_reg_ and very few CD4^+^CD25^+^Foxp3^−^ could be detected. Treatment with 2B010 resulted in a highly significant depletion of T_reg_ (Figure 6C). Few CD8^+^ T cells in the TIL expressed CD25, but production of granzyme B by CD8^+^ T cells was markedly enhanced in treated mice and was accompanied by a modest decrease in the level of CTLA-4 expression (Supplementary Figure 5A, 5B). Despite the presence of a high percentage of activated CD8^+^ T_conv_ cells capable of producing high levels of granzyme B, we did not observe any effect of 2B010 treatment on tumor growth (Figure 6D).

**Figure 7:**
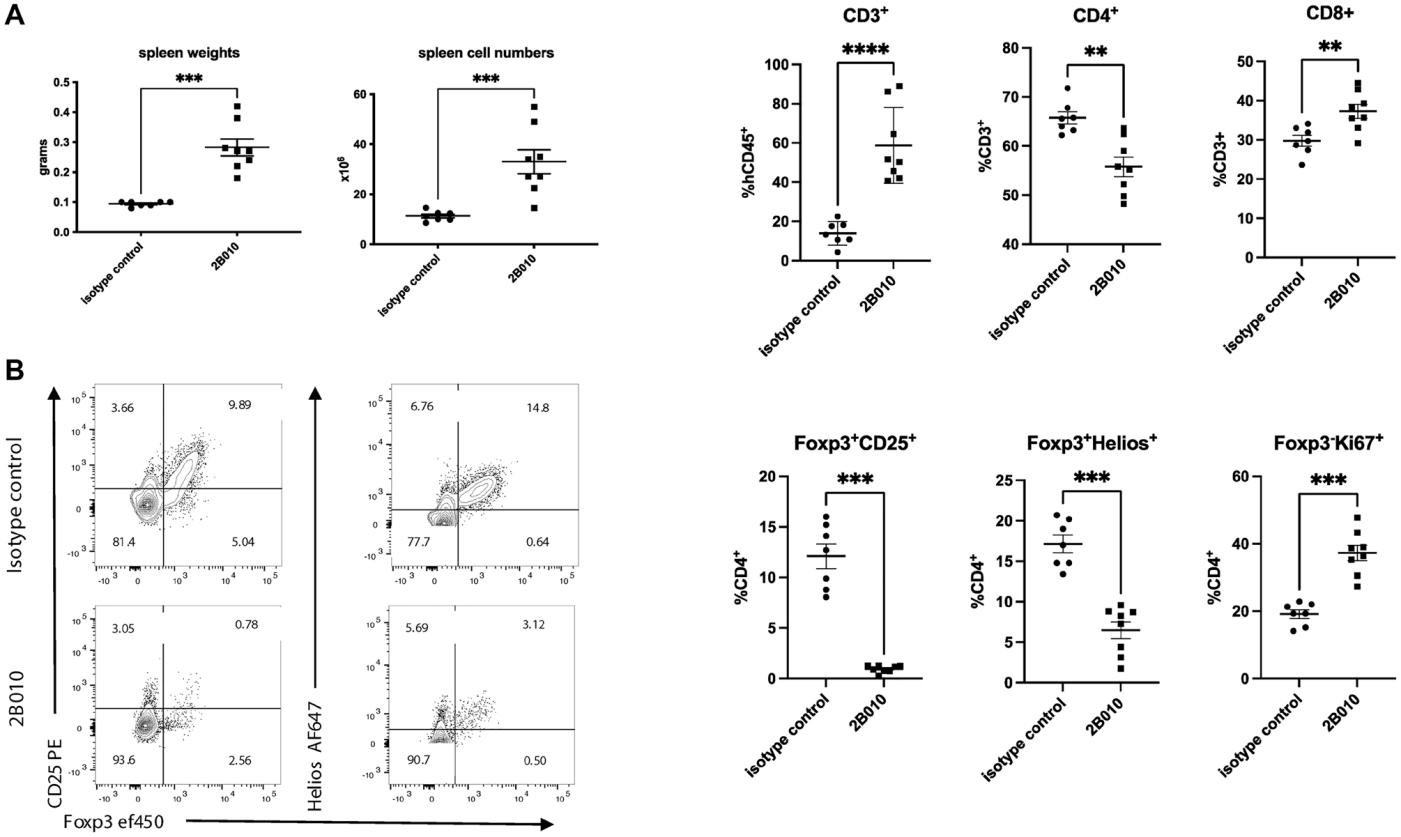
2B010 depletes T_reg_ from the spleen in tumor bearing mice. (**A**) Spleens from the same huCD34^+^ NSG mice used in Figure 6 which had been implanted with tumors and treated with either the 2B010 mAb or isotype control mAb were harvested on day 42. Spleen weights, cell numbers and the frequencies of CD3^+^, CD4^+^ and CD8^+^ T cells are shown. (**B**) Frequencies of CD4^+^Foxp3^+^CD25^+^, CD4^+^Foxp3^+^Helios^+^, as well as Ki-67 expression by CD4^+^Foxp3^−^ T cells are shown. *n* = 7–8 mice per group. Mann-Whitney statistical test was used. Data are shown as means ± SEM.

The effects of 2B010 treatment were not limited to the TME as 2B010 also resulted in an increase in total spleen weight, spleen cell numbers and an increase in the percentages of CD8^+^ T cells (Figure 7A). Treatment with 2B010 resulted in a marked decrease in CD4^+^Foxp3^+^Helios^+^ T_reg_ and moderate decrease in CD4^+^CD25^+^Foxp3^−^ T cells (Figure 7B). Marked increases in granzyme B producing, Ki-67^+^, CTLA-4^+^ and PD-1^+^ CD8^+^ T cells were observed which is consistent with a systemic response to the administration of 2B010 (Supplementary Figure 6A, 6B).

## DISCUSSION

We have identified an anti-CD25 mAb with several atypical properties which appears to have greater reactivity with activated T_reg_ cells than with activated CD4^+^ or CD8^+^ T cells. mAb 2B010 was selected because it bound to human T_reg_ cells which had been expanded *in vitro* for 7–14 days to a much greater extent than CD4^+^Foxp3^−^ T cells that had been similarly activated. More importantly, it also preferentially bound to human T_reg_ that had been activated *in vivo* during xeno-GVHD in NSG mice than to activated CD4^+^ T_conv_ cells. Chimeric 2B010 expressing wild-type human IgG1 Fc, but not a silent Fc, selectively deleted T_reg_ from mice with xeno-GVHD while sparing to a major extent activated CD4^+^CD25^+^Foxp3^−^ T_conv_ cells and CD8^+^CD25^+^ T cells. The degree of T_reg_ depletion could potentially be further improved by utilizing an Fc region that enhances ADCC and ADCP (such as a-fucosylated Fc) [32].

While it is widely accepted that activated CD4^+^Foxp3^−^ under the conditions used for expansion *in vitro* express elevated levels of CD25, the levels of CD25 expression on activated CD4^+^Foxp3^−^ T cells were somewhat less than the levels of CD25 expression on expanded T_reg_ when assayed with several of the therapeutic anti-CD25 mAbs including Clone D1 and basiliximab. In contrast, the difference in detection of CD25 on T_reg_ versus activated CD4^+^Foxp3^−^ T cells with 2B010 was roughly 100-1000-fold. Indeed, this finding raised the possibility that 2B010 was directed to another activation antigen. However, the level of reactivity of 2B010 to CHO cells transfected with human CD25 was like that seen with other anti-human CD25 mAbs. Thus, the reactivity of 2B010 to CD25 is complex as its reactivity with freshly explanted T_reg_, short-term expanded T_reg_, and short-term expanded T_conv_ cells is identical to that of standard anti-CD25 mAbs. Only after >6 days of stimulation *in vitro* does the difference in enhanced reactivity of 2B010 with T_reg_ compared to CD4^+^Foxp3^−^ T_conv_ cells become manifest. Taken together, it remains possible that 2B010 recognizes an epitope on CD25 that is present on freshly expanded T_reg_ or newly activated T_conv_ cells but is normally lost during longer activation of T_conv_ cells both *in vitro* and *in vivo*. It should be emphasized that the mature form of CD25 is a heavily glycosylated protein with a m.w. of ~ 50–55 kd while its protein backbone is only 30–35 kd. Unfortunately, our attempts to demonstrate that 2B010 recognized a carbohydrate epitope on CD25 were unsuccessful as were our attempts to define a specific binding site on soluble aglycosylated CD25. We did perform a limited series of competitive blocking studies using other anti-CD25 mAbs. The binding of Clone D1, basiliximab, 2A3, and BC96 which recognize the IL-2 binding site on CD25 was not blocked by 2B010. 2B010 also had no effect on IL-2 induced STAT5 phosphorylation or CD4^+^ T cell proliferation *in vitro* while both were blocked by Clone D1 further supporting the view that 2B010 does not recognize the IL-2 binding site.

Our studies using two different humanized mouse models have clearly elucidated a requirement for high levels of CD25 expression for 2B010 mediated T_reg_ deletion. In the xeno-GVHD model in which T_reg_ are activated and express very high levels of CD25, marked deletion of T_reg_ was observed following treatment of the mice with 2B010. In contrast, when NOG mice were reconstituted with CD34^+^ stem cells, the recipients did not develop xeno-GVHD and lower levels of CD25 expression were observed on T_reg_ and T_conv_ cells. The treatment of these mice with 2B010 resulted in transient modulation of CD25 expression without deletion of CD4^+^Foxp3^+^ T_reg_. We did not continue these studies for a long enough period to determine whether modulation of cell surface expression resulted in loss of T_reg_ secondary to the requirement for IL-2 for T_reg_ survival or whether modulation resulted in loss of T_reg_ suppressive function mediated by the ability of T_reg_ to compete with T_conv_ cells for IL-2 needed for T_conv_ cell function and expansion. These issues will be addressed in future studies. In any case, these models clearly illustrate the crucial role of the level of CD25 expression on the capacity of an anti-CD25 mAb to mediate deletion.

One difficulty encountered in the pre-clinical evaluation of an anti-human depleting mAb to augment tumor immunity is the choice of a suitable model to evaluate the effects of mAb treatment. The use of NSG mice that have been reconstituted with human PBMC and then given a transplantable tumor is frequently difficult to interpret as most of the anti-tumor effects may be secondary to xeno-GVHD rather than activation of tumor-specific T cells. Some PBMCs donors may be more highly reactive to mouse tissues than others and pre-selection of the donors is required further emphasizing the variability inherent to humanized mouse models.

NOG or NSG mice reconstituted with human cord blood CD34^+^ stem cells are cost prohibitive for extensive preclinical studies. In our experience, mice reconstituted with stem cells from a single donor preparation of cord blood cells may be quite variable in the extent of reconstitution and the distribution of different cell types (B cell predominate over T cells). In addition, the regulation of Helios expression appears to be abnormal in these mice as a high percentage of CD4^+^Foxp3^−^Helios^+^ cells could be detected. We selected an orthotopic breast cancer transplantation model for use in these mice. However, the CD4^+^ TIL in these mice were almost exclusively Foxp3^+^ Treg and very few CD4^+^Foxp3^−^CD25^+^ T cells were present. While treatment with 2B010 efficiently depleted Treg, we cannot claim that the depletion was selective due to the paucity of Foxp3^−^CD25^+^ cells in the TIL. Treatment with 2B010 also produced systemic effects and markedly depleted Treg from the spleen. In contrast to the results in the xeno-GVHD model where Foxp3^−^CD25^+^ were not depleted, we did observe significant depletion of Foxp3^−^CD25^+^ cells in the spleen of tumor bearing mice. Further testing of 2B010 in different preclinical models with different tumors in therefore indicated.

Solomon et al. [25] have proposed that an optimal anti-CD25 depleting mAb should be one that also fails to inhibit IL-2 binding to CD25 thereby allowing IL-2 to continue to augment the activity of primarily CD8^+^ cytotoxic cells and CD4^+^ cytokine producing T cells. This concept is consistent with the studies of Chinen et al. [33] which demonstrated that one mechanism for the suppressive function of Treg is to compete for IL-2 required for activation of CD8^+^, but not CD4^+^, T cells. 2B010 meets this criterion and in this regard, it closely resembles mAb RG6292 [23, 24]. It should also be noted that we selected mAb 2B010 from a group of 5 other mAbs that appeared to react with activated T_reg_ to a much greater extent than activated T_conv_ cells. These mAbs were like 2B010 as they failed to inhibit IL-2 binding to the IL-2R as assayed by their inability to block STAT5 phosphorylation. Similarly, two other anti-CD25 mAbs which do not recognize the IL-2 binding site and which have low affinity for CD25 were shown to have enhanced anti-tumor effectiveness *in vivo* [34, 35]. It remains to be determined whether there is a relationship between mAbs that do not inhibit IL-2 binding and preferential reactivity of the mAb with activated T_reg_ compared to activated T_conv_. A detailed analysis of the specific epitope on CD25 recognized by 2B010 by either x-ray crystallography or cryo-electron microscopy may also provide novel insights into its unique reactivity profile.

Taken together, our studies suggest that 2B010 represents an anti-CD25 mAb with unique properties in that it deleted T_reg_ from an inflammatory environment (GVHD) as well as from the TME. While our studies and those of Solomon et al. [25] using a mAb with similar properties clearly demonstrated the activation of CD8^+^ T_conv_ cells after deletion of T_reg_ from the TME, neither of the studies demonstrated effects on tumor size or potential for metastasis. The reason for this discrepancy is unclear but may be secondary to lack of sufficient tumor-specific T cells in both models and other uncharacterized limitations of the use of humanized mice for these studies. Both studies do strongly support the use of anti-CD25 mAbs for treatment of humans with malignancy either alone or in concert with check-point inhibitors.

## MATERIALS AND METHODS

### Mice

NOD.Cg-Prkdc^scid^IL2rg^tm1Wjl^/SzJ (NSG) mice were obtained from Jackson Laboratories and bred under NIAID contract with Taconic. NOD.Cg *Prkdcscid Il2rgtm1Sug* Tg(SV40/HTLV-IL3, CSF2) 10-7Jic/JicTac (NOG-EXL (hGM-CSF/hIL-3 NOG)) mice (TAC LINE 13395) CD34 engrafted or non-engrafted mice were obtained from Taconic. Animal protocols used in this study were approved by protocol LISB51 by the NIAID Animal Care and Use committee and are in accordance with the ARRIVE guidelines.

### *In vitro* expansion of T_conv_ and T_reg_

hPBMC were obtained from unidentified normal human blood bank donors. These studies are exempt from further ethical review by an Institutional Review Board and were performed according to guidelines of the National Institute of Allergy and Infectious Diseases. CD4^+^ T cells were enriched from hPBMC using the autoMacs^®^ Pro Separator (Miltenyi Biotec, Cat# 130-092-545) and then sorted for CD4^+^CD25^+^CD127^lo^ (T_reg_) and CD4^+^CD25^−^CD127^+^ (T_conv_). T_conv_ and T_reg_ were expanded using a 1:1 and 1:3 ratio, respectively, of DynaBeads Human T-Activator CD3/CD28 for T Cell Expansion and Activation (Cat# 11131D) to cells. For T_reg_, 300 U/mL of recombinant human IL-2 (TECIN, Hoffman-La Roche Inc.) was added to the culture while 30 U/mL was used for T_conv_. The cells were then cultured for 7–9 days. CD8^+^ T cells were purified with CD8 microbeads (Miltenyi Biotec, Cat no 130-045-201) and then further purified by cell sorting. CD8^+^ cells were expanded using the same conditions as CD4^+^ T cells.

### Generation of mouse anti-human T_reg_ mAbs

BALB/c mice were immunized by i.v. injection with expanded T_reg_ cells at multiple sites over a 25-day period (Supplementary Figure 1A). Hybridoma fusion(s) took place on Day 27. Hybridoma fusions were performed using isolated splenocytes and lymph node cells fused via PEG-1500 (Millipore) and SP2/0 mouse myeloma cells. Hybridomas were cultured for at least 12 days with 3 media exchanges. Media contained RPMI-1640, HAT Medium (hypoxanthine-aminopterin-thymidine medium), 10% FBS and Hybridoma Fusion and Cloning Supplement (Millipore). Supernatants were then harvested and tested for binding by indirect staining flow cytometry on expanded T_reg_ cells and expanded T_conv_ cells. Hybridomas supernatants with selective reactivity to expanded T_reg_ cells were then sub-cloned by limiting dilution and confirmed for enhanced binding to T_reg_.

### Assay of mAb affinity by surface plasmon resonance

Milligram quantities of antibody were purified by protein A affinity chromatography (GE Healthcare) and buffer exchanged into a 100 mM Histidine buffer. The affinity of the CD25 antibodies was determined by SPR using a Biacore^™^ 3000 surface plasmon resonance system captured on a CM5 sensor chip by an anti-human IgG prepared using the Biacore Human Antibody Capture Kit according to the manufacturer’s directions (GE Healthcare). Experiments were performed at 25°C using a 40 μl min−1 flow rate in Gibco 1X PBS Buffer w/o magnesium or chloride buffer. Recombinant IL-2R α-chain with a human IgG_1_ Fc-tag (Acrobiosystems, 1LA-H5251) 4 μg/ml was injected over the surface. Either clone D1 Fab or 2B010 Fab at 4, 2, and 1 μg/ml were immobilized on the chips. A 1:1 Langmuir binding model was used to fit all binding curves.

### Chimeric antibody generation

Hybridoma cells were isolated for antibody RNA recovery, sequencing and chimerization. Briefly, RNA was extracted from clonal hybridoma cells of interest, RT-PCR performed to produce cDNA and variable heavy and variable light mouse antibody genes amplified using a mouse Ig primer set. DNA gel electrophoresis confirmed each fragment. Subsequently, DNA vector ligation was performed, and cloning of V region amplicons followed by sequencing. Variable heavy and variable light sequences were subcloned into vectors containing the constant region of the human heavy and light chain, respectively. Vectors were designed for mammalian expression. An Fc-silenced human chimeric IgG_1_ was generated with two replacement mutations (Leu234A1a and Leu235A1a) that eliminate ADCC and CDC activity by reducing effector functions such as FcγR and complement binding [36]. Wild type (WT) indicates native mouse IgG Fc vectors used. The daclizumab sequence (PDP ID: 3NFP) was used to generate antibody via vectors and production as described above. This research antibody is referred to as daclizumab sequence produced antibody Clone D1.

### Flow cytometry

Cells (2−3 × 10^6^, splenocytes or PBMCs) were stained for analysis by flow cytometry. Human BD Fc block (564220) was used to block binding of antibodies to Fc receptors. A surface staining master-mix (50 mL) which included a live-dead dye (Invitrogen Live/Dead Fixable Near-IR Dead Cell Stain Kit) was prepared in PBS and added to each sample. Intracellular staining was done using the Invitrogen^™^ eBioscience^™^ Foxp3/Transcription Factor Staining Buffer Set (Invitrogen, cat 50-112-8857). Compensation was done using UltraComp eBeads (Invitrogen, Cat# 50-112-9040) and The ArC^™^ Amine Reactive Compensation Bead Kit (Invitrogen, Cat# A10346). The samples were acquired on either a BD Symphony or BD Fortessa which were calibrated each day with CST beads. FlowJo was used to analyze the sample data and isotype and FMO controls confirmed proper gating.

### Antibodies used for flow cytometry

Anti-human CD45 (HI30), anti-human IgG PE (HP6017), anti-human CD25 PE (MA251) which does not recognize the IL-2 binding site, anti-human Helios AF647 (22F6), anti-human CD45RO (UCHL1), anti-human CTLA-4 (L3D10, BNI3), anti-human Ki67 (Ki-67), anti-human PD-1 (EH12.2H7) were obtained from Biolegend. Anti-human CD3 (UCHT1), anti-human CD4(SK3), anti-human Ki67 (B56), were obtained from BD Horizon. Anti-human CD8 (SK1) was obtained from BD Optibuild. Anti-human CD25 PE (BC96) and A23 which recognize the IL-2 binding site on CD25 [29] and anti-human Foxp3 eF450 (236A/E7) were obtained from Invitrogen. Anti-human Granzyme B (GB11) and anti-human Ki67 (B56) were obtained from BD Pharmingen. Anti-human GITR (V27-580) was obtained from BD Biosciences. Anti-human pSTAT5 (47/Stat5(pY694)) was obtained from BD Phosflow. Anti-mouse Ig was obtained from Jackson ImmunoResearch.

### *In vitro* T cell proliferation and T_reg_ suppression assay

CD4^+^ cells were sorted as described above. HLA-DR^+^ cells were sorted from the CD4^−^ population using the autoMacs^®^. After sorting, cells were washed with cRPMI and counted. Sorted T conventional (T_conv_) cells were stained using the CellTrace^™^ Violet Cell Proliferation Kit (Invitrogen, Cat# C34557) and washed with cRPMI. Anti-CD3 (OKT3) antibody was added to HLA-DR^+^ cells to stimulate the cells. T_reg_ and Tconv were added at various concentrations along with the chimeric antibodies (20 μg/mL). Cells were stimulated for 4 days at 37°C, harvested and analyzed [37].

### *In vitro* STAT5 phosphorylation assay

Cells (1 × 10^6^) were transferred to FACS tubes along with 10 mg of each antibody. The tubes were incubated at 37°C, 5% CO_2_ for 20 minutes. 10U of IL-2 were added to the samples and incubated an additional 20 minutes. Cells were then fixed with 1 mL of pre-warmed BD CytoFix Fixation Buffer (Cat# BDB554655) and incubated for 10 minutes. The samples were permeabilized using BD Perm Buffer III (Cat# 558050) incubated on ice for 30 minutes. Cells were then stained for analysis by flow cytometry.

### *In vivo* studies

NSG mice were intravenously injected with 3 × 10^6^ hPBMCs. Spleens were harvested and analyzed by flow cytometry. In other experiments, 2B010 (400 μg) or an isotype control were injected intravenously. Spleens were then harvested, counted, and analyzed by flow cytometry. In certain experiments, CD34^+^ engrafted NSG or NOG EXL mice were treated with either 2B010 (400 μg) or an isotype control, and the number of T_reg_ in the blood were analyzed at indicated times. NSG mice were injected with expanded T_conv_ (3 × 10^6^) and treated with 2B010 (400 μg) or an isotype control 1-week post-injection. Spleens were harvested twelve days later and analyzed by flow cytometry.

### *In vivo* tumor studies

CD34^+^ engrafted NSG mice were injected orthotopically with 3 × 10^6^ MDA MB231– luc/GFP breast cancer cell line [38, 39] (Generously provided by L. Ridnour (NCI, NIH)). Starting on day 0, the mice received 400 μg of 2B010 or an isotype control weekly for six weeks. Mice were euthanized on day 42 after transplantation. Tumors were excised and placed in 1X HBSS digestion buffer (Lonza HBSS Cat# 10-508F; Sigma Aldrich Collagenase IV, Cat# C4-22-1G; Hyaluronidase; DNase IV (2.48 mg)) where they were cut into small pieces and then incubated on a shaker at 37°C for two hours. Tumor pieces were pressed through a filter and centrifuged at 50 g for 10 minutes at 4°C. Cells were washed and resuspended in 40% Percoll solution (Gibco DMEM Cat. # 11-960-044, Gibco 10X PBS Cat. # 10010-023, Millipore Sigma Percoll Cat. # P1644-1L). 80% Percoll solution was added to bottom of the tube, and the tube was centrifuged at 325 g for 23 minutes at 20°C. Spleens were also processed as previously described. Cells were washed and stained for FACS analysis.

### Statistical analysis

The FlowJo 10.8.1 software was used to perform the flow cytometry analysis. PRISM 9 (GraphPad software) was used to determine statistical significance. A Mann-Whitney *U*-test was used for all comparisons shown. Differences with *p* < 0.05 were considered statistically significant.

## SUPPLEMENTARY MATERIALS



## Abbreviations

ADCC: antibody dependent cellular cytotoxicity
ADCP: antibody dependent cellular phagocytosis
FACS: fluorescence activated cell sorting
GVHD: graft versus host disease
mAb: monoclonal antibody
PBMC: peripheral blood mononuclear cells
SPR: surface plasmon resonance
T_conv_: T conventional cells
TME: tumor microenvironment
T_reg_: T regulatory cell
