# Supplementary material for: A novel anti-human CD25 mAb with preferential reactivity to activated T regulatory cells depletes them from the tumor microenvironment
Source: Oncotarget. 2025 Jul 9;16:545–58. doi: 10.18632/oncotarget.28752 (PMC12243931; doi:10.18632/oncotarget.28752)
Supplement: Supplementary file 1 [file oncotarget-16-28752-s001.pdf]

A novel anti-human CD25 mAb with preferential reactivity to activated T regulatory cells depletes them from the tumor microenvironment

SUPPLEMENTARY MATERIALS

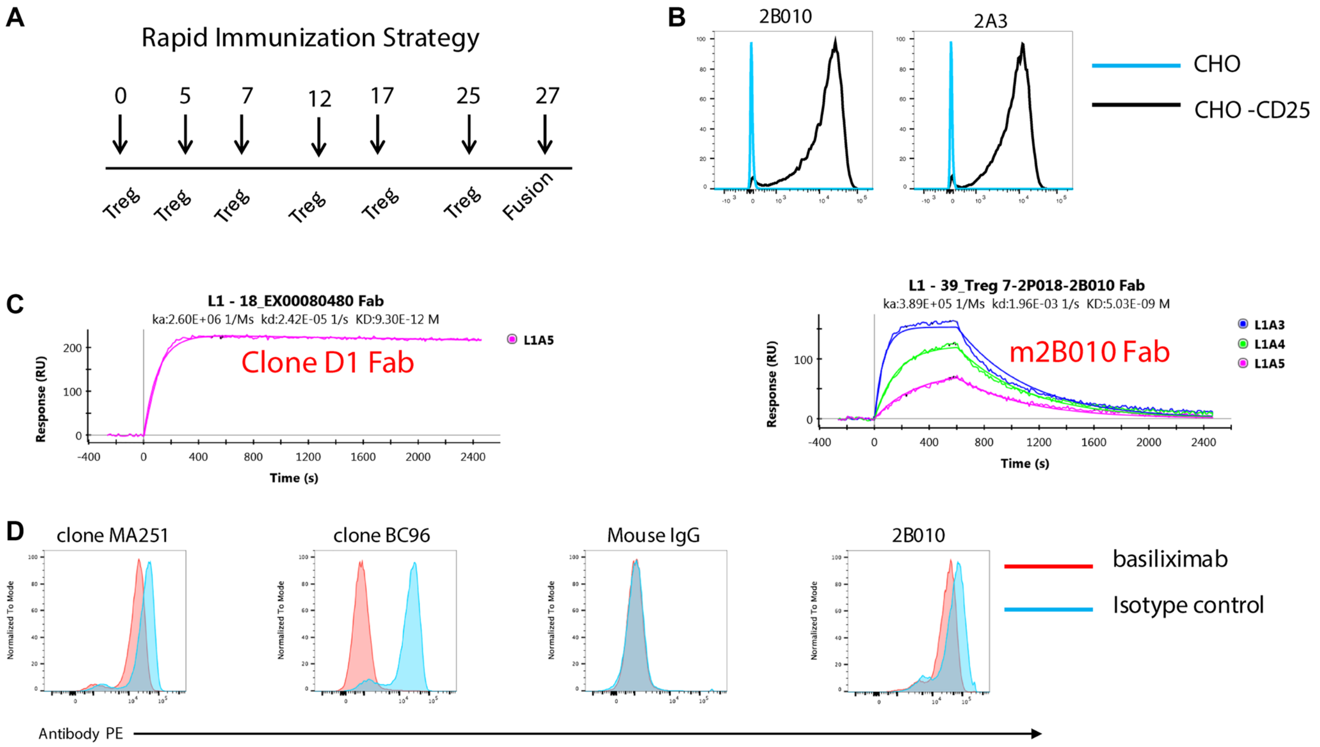

Supplementary Figure 1. (**A**) Immunization strategy. (**B**) Binding of 2B010 and 2A3 to hCD25 transfected or CHO cells. (**C**) SPR binding data – affinity. (**D**) Blocking experiment. Tregs were incubated with basiliximab or hIgG1 isotye control and then directly stained with MA251, BC96 or 2B010.

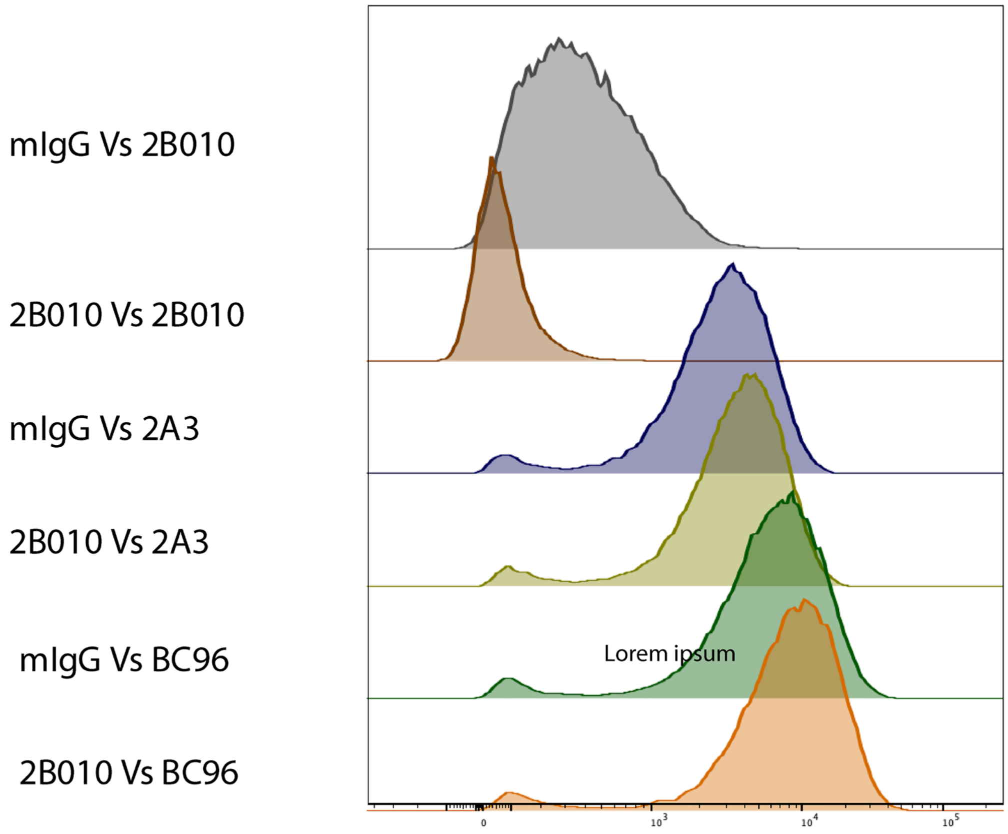

Supplementary Figure 2: 2B010 recognizes a unique epitope on Foxp3 $^+$Treg. CD4$^+$ T cells were isolated by negative selection with Miltenyi beads. Cells were incubated with saturating concentrations of mlgG or 2B010 for 30 minutes, washed and then stained with anti -FOXP3 and with PE -labeled 2B010, 2A3, or BC96. Results from gating on Foxp3 $^+$T cells.

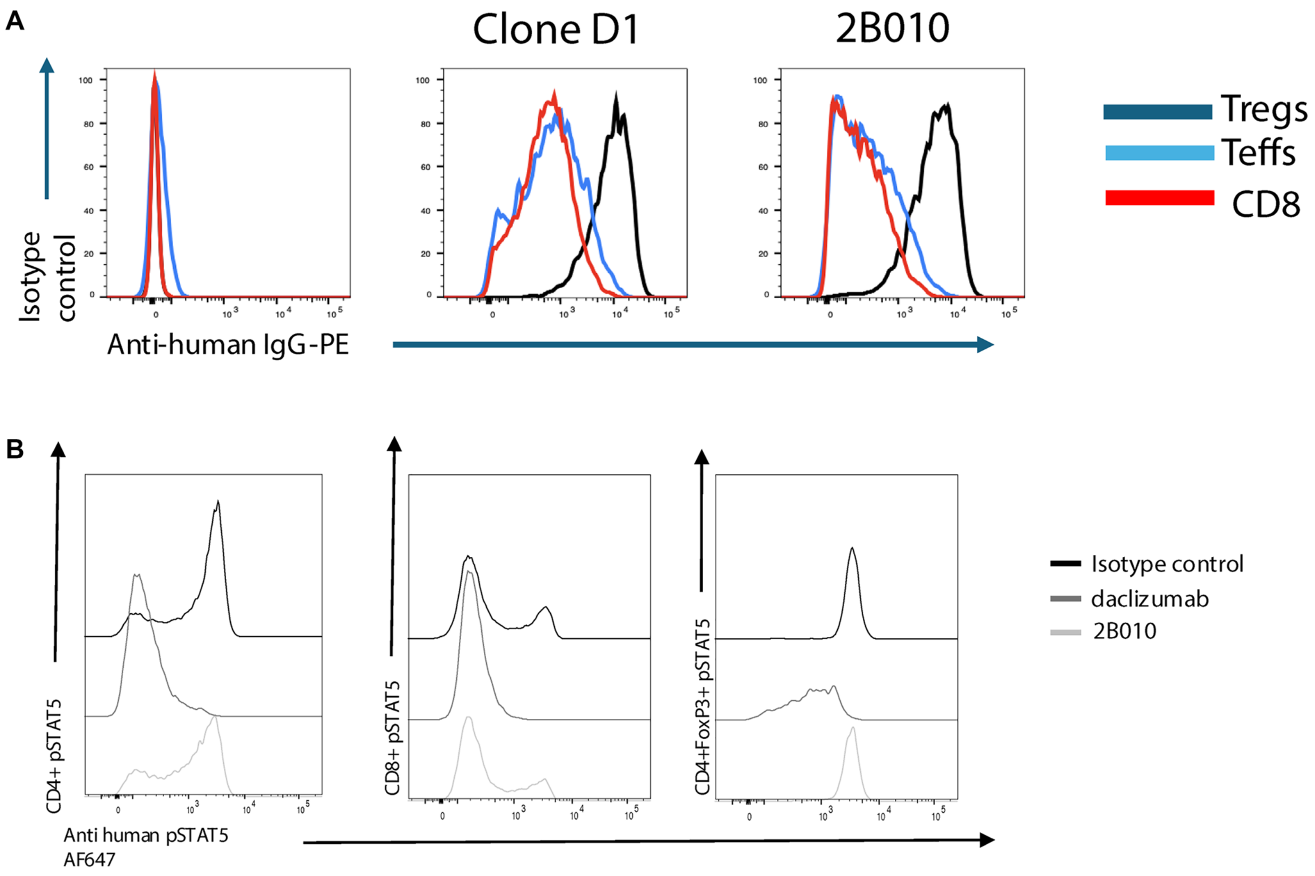

Supplementary Figure 3: (**A**) 2B010 preferentially reacts with T_{reg} expanded *in vitro*. $CD4^+CD25^+$, $CD4^+CD25^-$, and $CD8^+$ T cells were isolated from human PBMCs isolated from a second donor by cell sorting and stimulated with anti-CD3/CD28 beads and IL-2. Cells were stained with Clone D1 or 2B010 on day 7. (**B**) $CD4^+$, $CD8^+$ and $CD4^+Foxp3^+$ T_{reg} cells from a second donor were purified from PBMC by cell sorting, stimulated with IL-2 for 20 minutes in the presence or absence of isotype control mAb, clone D1, or 2B010. Cells were then washed and stained for pSTAT5.

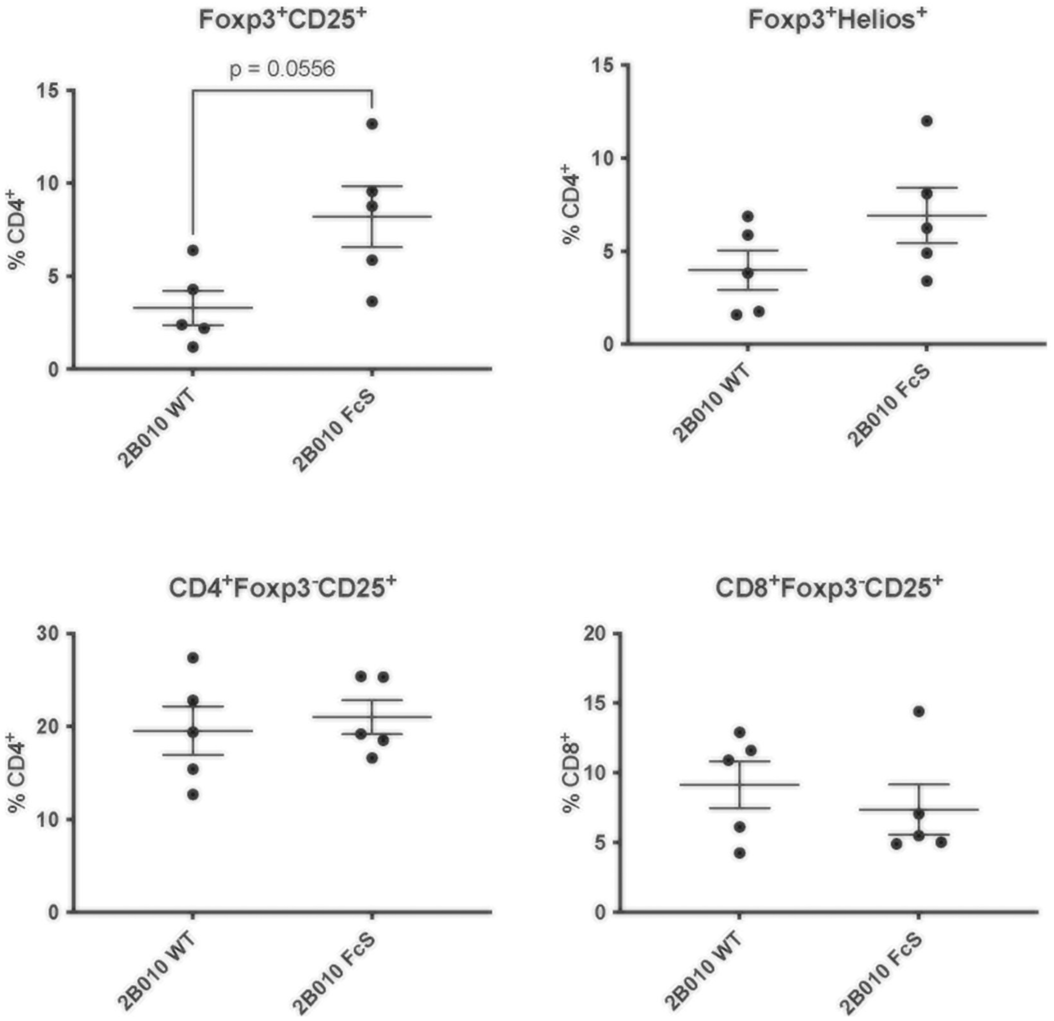

Supplementary Figure 4: NSG mice were reconstituted hPBMC and then treated with 2B010 WT or 2B010 Fe-silenced and frequencies of Tregs and CD8+T cells were analyzed on day 5 after treatment. *n* = 5 mice per group, Mann-Whitney statistical test was used. Data are shown as mean ± SEM.

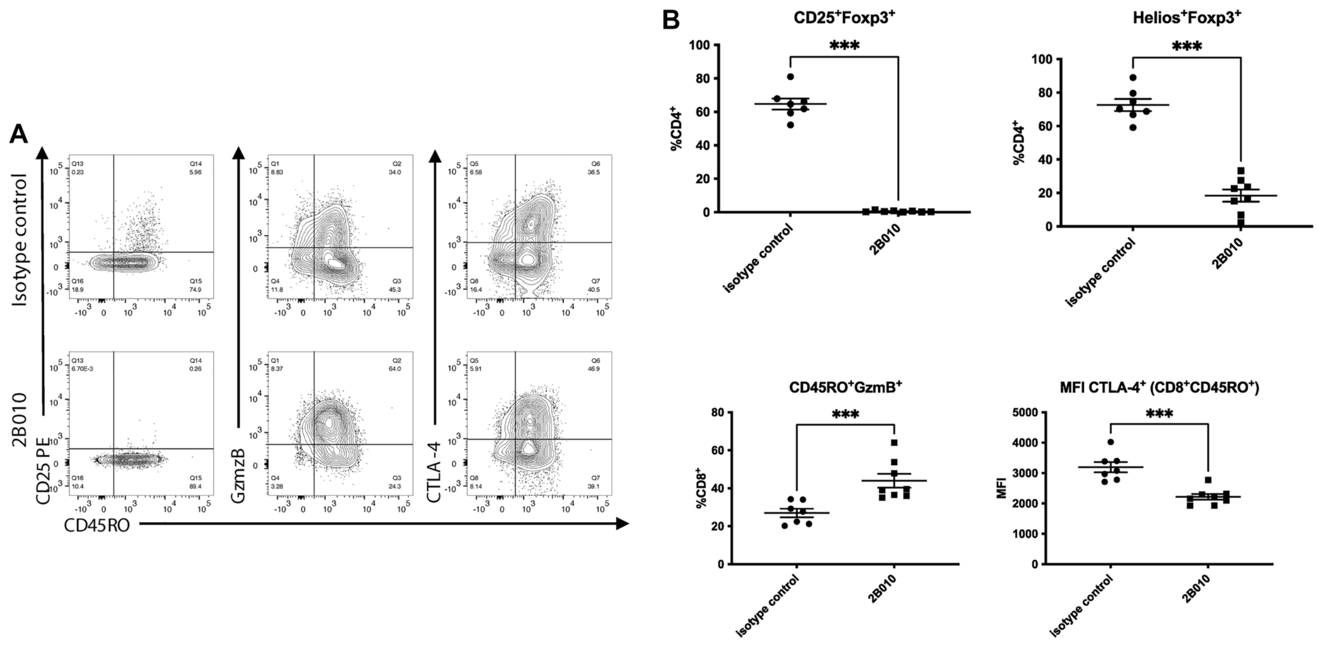

Supplementary Figure 5: (**A**) Expression of CD25, granzyme B, and CTLA-4 on memory (CD45RO^{+}) CD8^{+} T cells from TIL of mice treated with 2B10 and isotype control. (**B**) Summary data of the frequencies of CD25^{+}Foxp3^{+} T_{regs} and Helios^{+}Foxp3^{+} T_{regs} in the TIL of mice treated with 2B10 or isotype control mAb (top), frequencies ofCD8^{+}CD45RO^{+}granzyme B^{+} and the CTLA4 MFI on CD8^{+}CD45RO^{+} T cells shown in panel A. *n* = 7–8 mice per group. Mann-Whitney statistical test was used. Data are shown as means ± SEM.

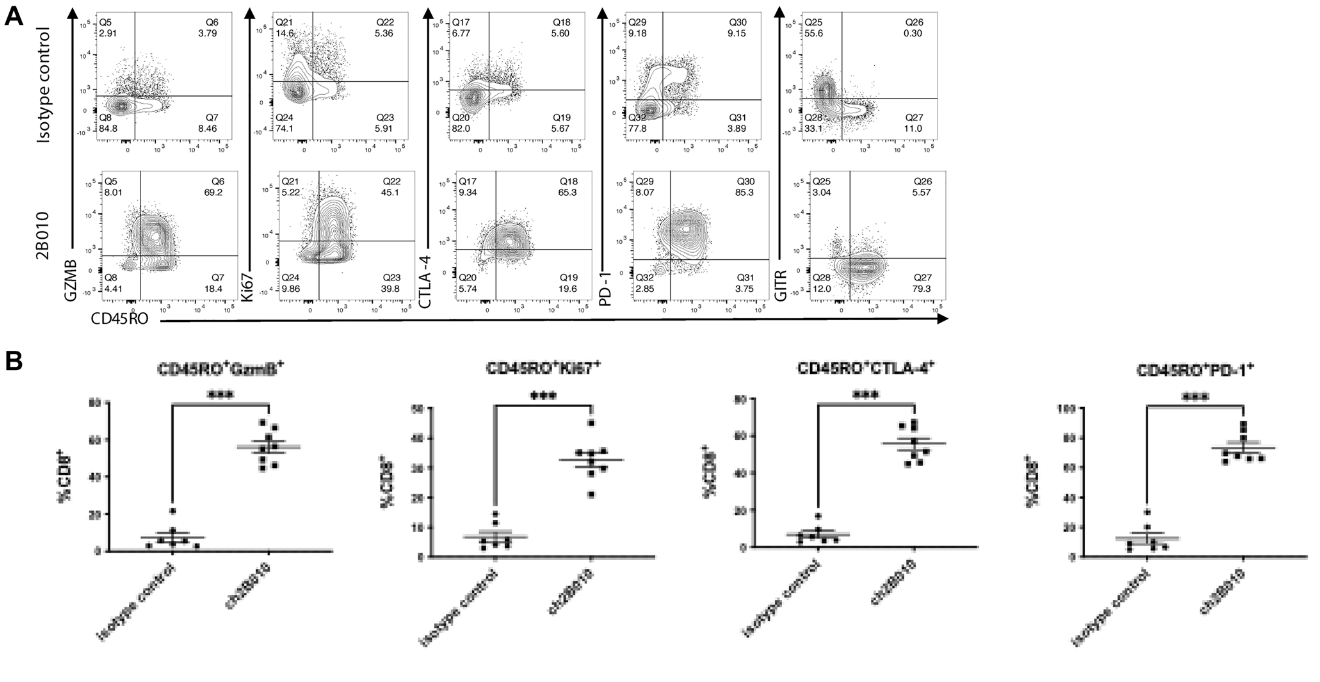

Supplementary Figure 6: (**A**) On day 0 huCD34 NSG mice were implanted orthotopically with 3 × 10^{6} MDA MB231 – luc/GFP cells and received 2B010 or isotype control weekly for total of 6 doses. At the end of the study (day 42), spleens were harvested and analyzed by flow cytometry. (**B**) Spleen CD8^{+} cells were stained for GzmB, Ki-67, CTLA4, and PD-1.
